# Sympatho–Vagal Dysfunction in Patients with End-Stage Lung Disease Awaiting Lung Transplantation

**DOI:** 10.3390/jcm9041146

**Published:** 2020-04-17

**Authors:** Eleonora Tobaldini, Gabriel D. Rodrigues, Giorgio Mantoan, Alice Monti, Giulia Coti Zelati, Camilla Cirelli, Paolo Tarsia, Letizia Corinna Morlacchi, Valeria Rossetti, Ilaria Righi, Mario Nosotti, Pedro Paulo da S. Soares, Nicola Montano, Stefano Aliberti, Francesco Blasi

**Affiliations:** 1 Department of Internal Medicine, Fondazione IRCCS Ca’ Granda Ospedale Maggiore Policlinico, 20122 Milan, Italy; eleonora.tobaldini@unimi.it (E.T.); giorgio.mantoan@studenti.unimi.it (G.M.); alice.monti@unimi.it (A.M.); giulia.coti@unimi.it (G.C.Z.); camilla.cirelli@unimi.it (C.C.); 2 Department of Clinical Sciences and Community Health, University of Milan, 20122 Milan, Italy; 3 Department of Physiology and Pharmacology, Biomedical Institute, Fluminense Federal University, Niterói 24210-130, Brazil; gabrieldias@id.uff.br (G.D.R.); ppssoares@id.uff.br (P.P.d.S.S.); 4 Respiratory Unit and Cystic Fibrosis Adult Center, Fondazione IRCCS Ca’ Granda Ospedale Maggiore Policlinico, 20122 Milan, Italy; paolo.tarsia@policlinico.mi.it (P.T.); letizia.morlacchi@policlinico.mi.it (L.C.M.); valeria.rossetti@policlinico.mi.it (V.R.); stefano.aliberti@unimi.it (S.A.); francesco.blasi@unimi.it (F.B.); 5 Department of Pathophysiology and Transplantation, University of Milan, 20122 Milan, Italy; 6 Thoracic Surgery and Lung Transplantation Unit, Fondazione IRCCS Ca’ Granda Ospedale Maggiore Policlinico, 20122 Milan, Italy; ilaria.righi@policlinico.mi.it (I.R.); mario.nosotti@unimi.it (M.N.)

**Keywords:** heart rate variability, cardiac autonomic control, lung transplantation, spectral analysis, symbolic analysis, cardiac autonomic dysfunction, autonomic nervous system

## Abstract

Although the literature demonstrates that cardiac autonomic control (CAC) might be impaired in patients with chronic pulmonary diseases, the interplay between CAC and disease severity in end-stage lung disease has not been studied yet. We investigated the effects of end-stage lung disease on CAC through the analysis of heart rate variability (HRV) among patients awaiting lung transplantation. Forty-nine patients on the waiting list for lung transplantation (LTx; 19 men, age 38 ± 15 years) and 49 healthy non-smoking controls (HC; 22 men, age 40 ± 16 years) were enrolled in a case–control study at Policlinico Hospital in Milan, Italy. LTx patients were divided into two groups, according to disease severity evaluated by the Lung Allocation Score (LAS). To assess CAC, electrocardiogram (ECG) and respiration were recorded at rest for 10 min in supine position and for 10 min during active standing. Spectral analysis identified low and high frequencies (LF, sympathetic, and HF, vagal). Symbolic analysis identified three patterns, i.e., 0V% (sympathetic) and 2UV% and 2LV% (vagal). Compared to HCs, LTx patients showed higher markers of sympathetic modulation and lower markers of vagal modulation. However, more severely affected LTx patients, compared to less severely affected ones, showed an autonomic profile characterized by loss of sympathetic modulation and predominant vagal modulation. This pattern can be due to a loss of sympathetic rhythmic oscillation and a subsequent prevalent respiratory modulation of heart rate in severely affected patients.

## 1. Introduction

Lung transplantation (LTx) is a reliable lifesaving option for selected individuals with end-stage pulmonary disease. Although the number of LTx is increasing over time, the mortality during the waiting period is still high [1].

One of the possible complications in patients on the waiting list for LTx are cardiovascular events, which also could impact the mortality rate. Specific hemodynamic factors predict risk in patients awaiting LTx, suggesting a higher organ allocation priority [1]. However, the factors that impact the outcome of patients referred for LTx still need to be elucidated [2].

Heart rate variability (HRV) is a reliable and non-invasive tool able to provide information on the autonomic nervous system control of cardiovascular functions [3]. A reduction of global HRV has been associated with poor prognosis in several cardiovascular diseases [4]. Although cardiac autonomic modulation seems impaired in pulmonary diseases [5,6,7], the interplay between cardiac autonomic modulation and severity of end-stage pulmonary disorders in patients waiting for LTx has not been studied yet.

The Lung Allocation Score (LAS) has been developed by the United Network for Organ Sharing as a tool to allocate donated lungs according to a ‘net benefit’ concept. The LAS estimates each patient’s medical urgency prior to transplantation and success probability after surgery, in order to ensure higher priority to patients who could benefit more from lung transplant. The score ranges from 0 to 100 and is calculated through an algorithm which considers each patient’s disease diagnosis, physical characteristics, and clinical data. Patients with higher LAS will receive higher priority for transplant [8]. We used LAS to assess our patient’s disease severity, in terms of ranking priority, urgency of surgical intervention, and success probability. Our Transplant Center adopted the Eurotransplant International Foundation LAS Calculator [9].

We formulated the hypothesis that lung disease severity—no matter the disease—may be related to cardiac modulation markers through HRV analysis. This study aimed at (1) assessing cardiac autonomic modulation in a population of patients with end-stage lung disease who are candidate for LTx in comparison with healthy controls and (2) testing the hypothesis that disease severity may be associated with specific cardiac autonomic markers using two different tools, spectral and symbolic analysis, for the evaluation of cardiac autonomic modulation.

## 2. Materials and Methods

### 2.1. Sample

This was a case–control, prospective study. The protocol was approved by the Internal Review Board of Ospedale Maggiore Policlinico, Fondazione IRCCS Ca’ Granda, Milan, Italy (protocol number 181, January 2017, 749-2016 bis) and was developed in accordance with the Declaration of Helsinki. All the subjects signed an informed written consent before study participation.

From January 2017 to June 2018, consecutive adults on LTx waiting list referring to the Lung Transplant Program of the Policlinico Hospital, Milan, Italy, were enrolled. A cohort of healthy non-smoking controls were enrolled as well, matched for sex and age with the cases.

The absence of a stable sinus rhythm on electrocardiogram (ECG), percentage of extrasystoles >5% on ECG, pacemaker rhythm, non-invasive mechanical ventilation, and ongoing exacerbations (exacerbation of chronic obstructive pulmonary disease, bronchitis, pneumonia) and/or hospitalization were considered exclusion criteria for this study.

### 2.2. Experimental Design

All subjects underwent the recoding of ECG and respiration using a thoracic piezoelectric belt in supine (SUP) position for 10 min and during active standing for 10 min, for a total length of 20 min of recording per patient. Cardiovascular variables were acquired using an ad hoc telemetric system device (BT 16 Plus, Francesco Marazza Elettronica, Monza, Italy). During the recordings, the subjects were in spontaneous breathing but they were not allowed to talk.

The patients’ respiratory function was also assessed through a 6 minute walking test (6MWT) to examine their physical capacity status [10]. Spirometric tests were also performed, taking as satisfactory the highest measurement of forced vital capacity (FVC) and forced expiratory volume in 1 sec (FEV1) [11]. The diffusing capacity of the lung for carbon monoxide (DLCO) was also estimated.

### 2.3. Lung Disease Severity and Autonomic Modulation

In order to compare autonomic regulation among LTx patients, the LAS was used to assess disease severity. The median LAS value (33.8) and the 75th percentile LAS value (37.8) were used as thresholds to categorize patients into three groups: (a) less severe disease (lower than or equal to the median); (b) severe disease (between 50th and 75th percentile); (c) very severe disease (above the 75th percentile). Autonomic regulation parameters were compared by linear and non-linear methods of analysis.

### 2.4. Heart Rate Variability Analysis

The HRV was evaluated through a specific software (Heart Scope II, Amps LLC, New York, NY, USA) on short samples of 300 beats at rest and orthostatism (ORT) [12]. Every 10 min, a stable segment of 300 beats associated with stable breathing was selected from the tachogram, whose length allows for an adequate spectral resolution [13]. To evaluate the autonomic dynamic response to ORT, we calculated the ∆ORT% ((HRV in SUP position − HRV in ORT position)/HRV in SUP position).

Spectral analysis was performed through the autoregressive model, with a Hanning window and 50% overlap to obtain the spectral power in the low-frequency (LF, frequency band bounded between 0.04 and 0.15 Hz) and high-frequency (HF, frequency band bounded between 0.15 and 0.40 Hz and synchronous with respiration) components. The model order was estimated for each segment by the Akaike’s criterion in the present data bounded between 5 to 14 [12]. The LF and HF components can also be expressed in normalized units (LFnu and HFnu). The normalized components were obtained by dividing each band power by the total power after subtracting the very low component (<0.04 Hz). The autonomic balance was calculated as the LF/HF ratio [14].

Nonlinear dynamics were evaluated by symbolic analysis. The R-R dynamics were classified into 3 patterns families: (a) patterns with no variation (0V; all 3 symbols were equal); (b) patterns with 1 variation (1V; 2 consequent symbols were equal, and the remaining symbol was different); and (c) patterns with 2 variations (2UV or 2LV; all symbols were different from the previous one). The patterns were expressed as percentages. From a mathematical point of view, the class 1V might be found but has no significant association with any autonomic tests used to validate the method [15]. The class 0V is considered a marker of sympathetic modulation, while 2LV and 2UV are markers of vagal modulation [16].

The relationship between heart frequency and respiration rate was also assessed to compare our patients. Through cross-spectral analysis, we calculated the squared coherence function (k^2^) at high frequencies between the respiratory rate and the heart rate for every patient. RR-RESP HFk^2^ could range between 0 (perfect uncorrelation) and 1 (perfect correlation).

### 2.5. Statistical Analysis

The Shapiro–Wilk test was used to evaluate the normality of the samples. Data are reported as median and interquartile range (IQR 25%–75%). HRV index comparisons between the groups were performed by unpaired t-test (*p* < 0.05) or by Mann–Whitney U test if the data were not normally distributed. Pearson’s correlation was used to test the relantionship between heart rate, heart rate variability, and symbolic analysis variables.

GraphPad Prism version 7.0 (GraphPad Software Inc., San Diego, CA, USA) and SigmaPlot 12.0 (Systat Software Inc., San Jose, CA, USA) were used.

## 3. Results

### 3.1. Study Populations

A total of 49 patients on the waiting list for LTx (Group LTx; 30 women; mean (SD) age: 38 ± 15 years) and 49 healthy non-smoking controls (Group HC; 27 women; mean (SD) age: 40 ± 16 years) were examined. Among the LTx Group, 32 patients were diagnosed with cystic fibrosis, 5 with idiopathic pulmonary fibrosis, 3 with chronic obstructive pulmonary disease, 2 with nonspecific interstitial pneumonia, 2 with systemic scleroderma, and 5 with other indications for LTx. Demographic characteristics, cardiovascular risk factors, respiratory function, chronic lung infections, and medications of patients on the lung transplant list are presented in Table 1. HC had no history of cardiopulmonary disease and did not take any chronic medication.

### 3.2. Autonomic Evaluation of End-Stage Lung Disease Patients vs. Healthy Controls

LTx patients were characterized by higher HR and, as regards spectral analysis, lower TP, with a reduction of total heart rate variability (Table 2, column (a)). As to sympatho–vagal balance, LTx patients showed higher levels of LFnu, higher LF/HF, and lower levels of HFnu than healthy controls. These data suggest a lower vagal modulation and a higher sympathetic modulation in patients with end-stage lung disease in comparison to healthy controls. The respiratory rate was significantly coupled with the HF component of the RR interval, as expressed by RR-RESP HFk^2^ values (> 0.5) for all groups (Table 2 and Table 3); LTx patients showed higher levels of RESP HF than HC.

**Figure 1:**
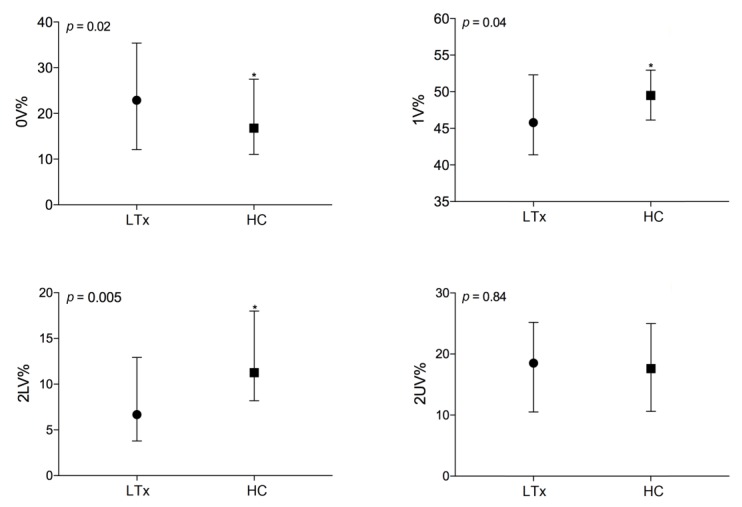
Comparison of autonomic parameters evaluated by symbolic analysis between healthy controls (HC, *n* = 49) and patients on the waiting list for lung transplant (LTx, *n* = 49) in supine position; *, statistically significant.

Symbolic analysis revealed higher levels of 0V% (23 (12–35) vs. 16 (11–27), *p* = 0.02), a marker of sympathetic modulation, and lower levels of 2LV% (7 (4–13) vs. 11 (8–18), *p* = 0.005), a marker of vagal modulation, in comparison to healthy controls (Figure 1). These observations are consistent with the previous ones, confirming a higher sympathetic modulation and a lower vagal modulation in patients with end-stage lung disease. In the present study, patient’s average heart rate and 2UV were negatively correlated in supine position, as the faster the HR, the lower the 2UV (*r* = −0.29, *p* = 0.04), and negative correlation was observed also with HF spectral power (*r* = −0.30, *p* = 0.03), suggesting less variability and lower vagal modulation of the heart explored in the time domain, spectral analysis, and symbolic analysis.

### 3.3. Autonomic Evaluation Among End-Stage Lung Disease Patients According to Disease Severity

The results of the spectral analysis are reported in Table 3, column (a). Patients with a LAS lower than or equal to the median LAS showed higher LFnu than patients with a LAS above the median. No other significant differences were found in HR and at spectral decomposition when comparing these two groups of patients.

**Figure 2:**
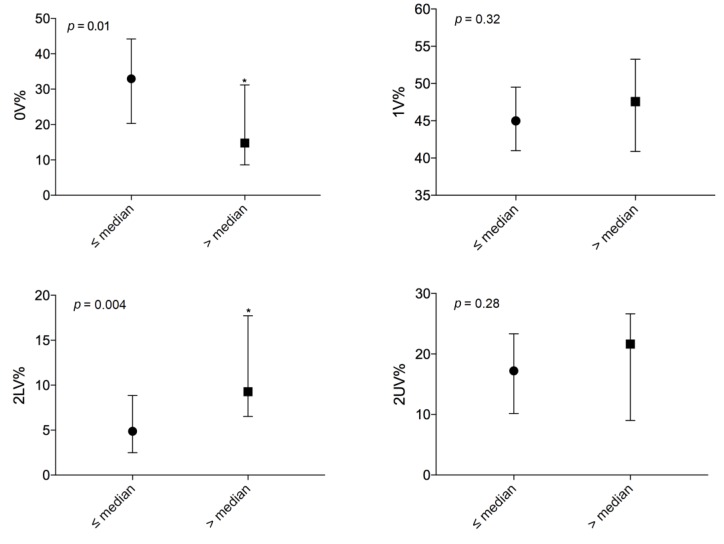
Comparison of autonomic parameters evaluated by symbolic analysis between patients on the lung transplant list with a LAS lower than or equal to the median (*n* = 25) and patients on the transplant list with a LAS above the median (*n* = 24); *, statistically significant.

When performing a symbolic analysis (Figure 2), a difference was found between the two groups: more severely affected patients had significantly lower levels of 0V% (20 ± 16 vs. 31 ± 15, *p* = 0.01) and higher levels of 2LV% (10 (7–18) vs. 5 (3–8), *p* = 0.004).

These data suggest a lower sympathetic modulation and a predominant vagal modulation in more severely affected patients.

### 3.4. Comparison Above and Below the 75th Percentile LAS

The results of the spectral analysis are reported in Table 3, column (b). Spectral decomposition revealed lower levels of LFnu for patients with a LAS above the 75th percentile in comparison with patients with a LAS lower than or equal to the 75th percentile. More severely affected patients also showed a lower LF/HF ratio in comparison with less severely affected ones. No other significant differences were found in HR and at spectral decomposition.

**Figure 3:**
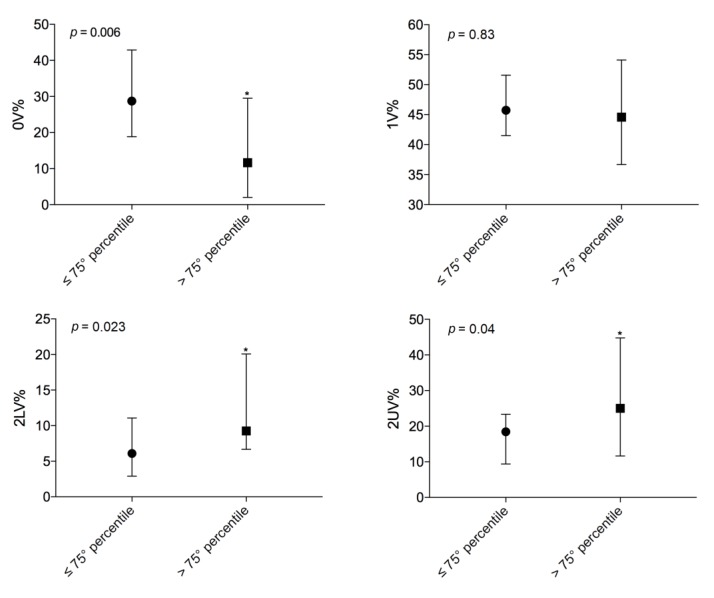
Comparison of autonomic parameters evaluated by symbolic analysis between patients on the lung transplant list with a LAS lower than or equal to the 75th percentile (*n* = 37) and patients on the transplant list with a LAS above the 75th percentile (*n* = 12); *, statistically significant.

Again, symbolic analysis (Figure 3) showed a difference between the two groups: more severely affected patients had lower levels of 0V% (15 (±13) vs. 29 (±15), *p* = 0.006) and significantly higher levels of 2LV% (11 (7–19) vs. 6 (3–11), *p* = 0.023) and 2UV% (26 (12–37) vs. 18 (10–23), *p* = 0.04). These data confirmed a lower sympathetic modulation and a predominant vagal modulation in more severely affected patients.

### 3.5. Cardiac Autonomic Control during the Active Standing Test

We also assessed the autonomic dynamic response to ORT by comparing healthy controls and patients waiting for LTx. The ∆ORT% of both spectral and symbolic parameters were compared; the results are reported in Table 2, column (b).

LTx patients showed a significantly lower ∆ORT% in HR, LFnu, HFnu, and LF/HF, revealing a reduced variation of autonomic modulation with the active standing test in comparison to HC and a different sympatho–vagal balance. The results of the symbolic analysis also showed stronger variations in 0V% (marker of sympathetic modulation) and in 2UV% (marker of vagal modulation) in healthy controls than in patients with end-stage lung disease (∆ORT% 0V%: 97 (30–244) vs. 38 (−6–105), *p* = 0.014; ΔORT% 2UV%: −58 (−72–−16) vs. −18 (−47–28), *p* = 0.001).

## 4. Discussion

The major findings of the present study are: (1) Patients with end-stage lung disease have an autonomic imbalance in the direction of higher sympathetic and lower vagal modulation of the heart at rest and blunted cardiac autonomic responses to orthostatic stress when compared with a healthy control group; (2) Patients with more severe lung disease assessed by LAS showed an autonomic profile characterized by a prevalent cardiac vagal respiratory modulation and a loss of sympathetic efference compared to less severe lung disease patients.

Patients with cardiac autonomic dysfunction present several associated lung diseases, such as chronic obstructive pulmonary disease (COPD) [6,7], pulmonary arterial hypertension (PAH) [17], and cystic fibrosis (CF) [18] compared to age-matched controls. Although global HRV is reduced in patients with several lung diseases compared to healthy controls, the sympatho–vagal balance may be different considering disease severity and respiratory function [5,6]. The results of the current study indicate that in patients with end-stage lung disease there is a prominent reduction in cardiac autonomic modulation in the direction of vagal control and an increase in sympathetic modulation in comparison to age- and sex- matched healthy individuals.

Airflow obstruction levels in COPD increase the risk of ventricular arrhythmia and are associated with worse linear and non-linear heart rate dynamics [6]. Impaired HRV responses are associated with disease severity, although diverse pathophysiological manifestations may be present due to the complexity of COPD as a disorder [6,7]. For instance, a worse oxygenation status in COPD patients is associated with higher cardiac vagal and lower cardiac sympathetic outputs. Although there is no direct relation with the degree of airway narrowing in cardiac autonomic modulation, chronic hypoxemia can increase cardiac vagal activity and depress the sympathetic modulation of COPD patients [7]. Recently, a similar cardiac autonomic profile was reported in patients with community-acquired pneumonia (CAP) [5]. The global contribution from cardiac autonomic control, represented by the total power index of HRV spectral analysis, was reduced in CAP patients in comparison with healthy controls. Symbolic analysis showed that vagal-mediated HRV was higher and the sympathetic rhythmical component was reduced in CAP patients compared to healthy controls. Also, HRV sympathetic oscillations were particularly reduced in more severely affected patients, i.e., patients with a time to clinical stability >7 days, compared to less severely affected patients (time to clinical stability ≤7 days).

The current study demonstrated that patients with more severe end-stage lung disease (as measured by LAS) had an autonomic profile characterized by a predominant respiratory vagal modulation and a loss of sympathetic rhythmic oscillation. Furthermore, patients with more-advanced-stage lung disease showed a global reduction of HRV, with a loss of sympathetic oscillatory heart rhythmicity, while maintaining vagal respiratory modulation. On the other hand, in less severe stages of the disease, sympathetic modulation prevailed in response to a series of pathological mechanisms (such as hypoxia). Taken together, these are the potential underlying mechanisms to explain the HRV relationships with lung disease severity in end-stage lung disease patients.

According to previous studies, pulmonary hemodynamics are a predictor of mortality among patients awaiting LTx. For instance, patients on the LTx waiting list who have increased pulmonary vascular resistance (PVR) and a lower force vital capacity (FVC %) should be considered for a higher organ allocation priority [1]. We suggest that cardiac autonomic control (CAC) assessed by HRV could provide new insights into pulmonary hemodynamics regarding cardiovascular risk and higher organ allocation priority in patients awaiting LTx.

For example, in PAH, sympathetic overactivity is evidenced in some studies by increased systemic vascular resistance [19] or direct measurement of muscle sympathetic nervous activity, and chronic overactivity could relate to disease severity [20,21]. However, HRV studies revealed that, regarding the autonomic modulation of the heart, both sympathetic and vagal branches are impaired in PAH patients compared to healthy controls [17,21,22]. The incoherence of increased muscle sympathetic nerve activity and decrease in both vagal- and sympathetic-mediated HRV indexes is not completely explained yet. However, a reasonable underlying mechanism maybe similar to the one previously reported for heart failure, where the LF power of the HRV spectral analysis seems related to a reduced susceptibility to respond to changes in sympathetic tone [23,24]. Thus, the reduction in LF power in PAH patients may result from chronic sympathetic activation, as has been reported previously [21].

Most of the patients (65%) included in the current study had CF. This complex disease affects many organs, but the associated autonomic nervous system regulations have not yet been widely studied [18]. Although autonomic nervous system disruption may contribute to CF pathogenesis, there is little knowledge of the cardiac autonomic profile of CF patients. To our knowledge, there is a single study that investigated the cardiac autonomic profile in adults with CF, which did not find any differences in HRV markers between CF patients and healthy controls. However, in the CF group, FEV1 values (as percentage of predicted values) were positively associated with the cardiac sympatho–vagal balance (LF/HF) [25]. This may be explained by a physiological afferent vagal nerve activation with larger inspiratory tidal volumes related to pulmonary disease progression. In children with CF, a predominance of cardiac sympathetic modulation to vagal modulation was found in comparison with age-matched healthy controls [26]. Neither Szollosi et al. [25] nor Florêncio et al. [26] investigated patients with age, clinical features, or disease severity similar to those of the patients in our study, which complicates comparisons with our findings.

The predominance of CF in our case study is mainly due to the specialization of our Transplant Center. We considered the hypothesis of a bias of our study towards CF. We conducted analyses on subgroups comparing CAC and respiratory characteristics of patients affected by CF or diagnosed with other pathologies. We included comparisons between both whole groups and LAS-restricted sub-groups, but we did not find any difference (see Supplementary Materials, Tables S1, S2 and S3 for sub-groups analyses). Thus, we could speculate that the results are not related to the underlying disease. However, on this topic a cautious approach should be adopted, and we cannot rule out the existence of different autonomic patterns in end-stage lung diseases of different origins.

HRV markers at rest provide relevant information regarding cardiac autonomic modulation. However, the vagal and sympathetic contributions to cardiac regulation could be highlighted by the HRV response to an autonomic stimulus, such as active standing test (orthostatic stress) [14,16,27]. The results of the current study indicate that the sympathetic- and vagal-mediated HRV responses (∆LF and ∆HF, respectively) to ORT stress are blunted in end-stage lung disease patients in comparison to healthy controls. Recently, moderate to severe COPD patients showed an impaired HRV response to orthostatic stress and breathing maneuver, which was not present in less severely affected patients [6]. Although the HRV response to active standing test has been useful to add novel information regarding the association between cardiac autonomic regulation and cardiovascular outcomes in some disease conditions [16,27], the role of HRV analysis in relation to autonomic maneuvers must be further elucidated in patients with pulmonary disease.

In interpreting our results, some limitations must be taken into account. First, 49 patients and 49 healthy controls were included in this study; although our sample is sizable compared to other studies in this research area, further developments and more significant data could be achieved through an expansion of the sample size. Second, as we previously explained, the Lung Allocation Score was not implemented to provide a direct measure of disease severity: it has been developed to assess patients’ medical urgency, need for surgery, and survival probability after transplantation, and with this mind, we used it also to assess illness severity. Third, the population had a prevalence of cystic fibrosis and, although we conducted promising sub-groups analysis, this must be considered a limitation when interpreting our results. Finally, we did not exclude patients on different medications such as anticholinergics, beta blockers, and beta-agonists, which could affect cardiac autonomic control. However, this study aimed to characterize patients with end-stage lung disease in real-life conditions, and we know very well that many patients in these conditions are on medications affecting HRV. This element must be considered a limitation when interpreting the study results.

## 5. Conclusions

End-stage lung disease patients awaiting lung transplantation show higher sympathetic- and lower vagal-mediated HRV at rest and blunted HRV responses for both vagal and sympathetic indexes to orthostatic stress compared to age-matched healthy controls.

Furthermore, a prevalence of cardiac vagal modulation compared to sympathetic modulation is present in more severe lung disease patients compared to less severe lung disease patients, as assessed by the Lung Allocation Score.

From the clinical point of view, these results could be used by clinicians for a better stratification of LTx patients, considering that more severely affected patients have an autonomic profile characterized by loss of sympathetic modulation and predominant vagal modulation, which could be related to a worse prognosis. These data, if confirmed on a larger population, will help clinicians better stratify patients and identify those at high risk. 

## Figures and Tables

**Table 1:** Demographics, cardiovascular risk factors, respiratory function, chronic lung infections, and medications of patients on the lung transplant list.

	Study Population *n* = 49
**Demographics, *n* (%)**	
Age, mean (SD) years	38 (± 15)
Female	30 (61)
Body mass index, mean (SD)	21.8 (± 3.8)
Lung Allocation Score, median (IQR)	33.777 (32.4–37.5)
Cystic fibrosis	32 (65.5)
Idiopathic pulmonary fibrosis	5 (10)
Chronic obstructive pulmonary disease	3 (6)
Nonspecific interstitial pneumonia	2 (4)
Systemic scleroderma	2 (4)
Other indications for lung transplant	5 (10)
**Cardiovascular risk factors, *n* (%)**	
Hypertension	6 (12)
Diabetes	20 (41)
Pulmonary hypertension	12 (24.5)
mPAP, mean (SD) mmHg	22 (± 5)
**Respiratory function, median (IQR)**	
Exacerbations, *n* (%)	32 (65.5)
P/F, mean (SD)	305 (± 61)
pCO2, mmHg	42 (38–48)
FEV1 % of predicted	31 (24–49)
FVC % of predicted, mean (SD)	54 (± 16)
FEV1/FVC ratio, mean (SD)	60 (± 21)
DLCO, mean (SD) %	45 (± 22)
6MWT, mean (SD) m	406 (± 169)
**Chronic lung infections, *n* (%)**	
*Burkholderia cepacia*	1 (2)
*Pseudomonas aeruginosa*	27 (55)
MRSA	9 (18.5)
*Aspergillus*	8 (16.5)
*Candida albicans*	3 (6)
NTM	3 (6)
**Medications, *n* (%)**	
Beta-blockers	7 (14.5)
Beta-agonists	37 (75.5)
Anticholinergics	12 (24.5)
Endothelin Receptor Antagonists	1 (2)
Prostanoids	2 (4)
PDE5 inhibitors	1 (2)
Steroids	40 (81.5)
Oxygen	37 (75.5)

*n*, number; SD, standard deviation; IQR 25–75, interquartile range; mPAP, mean pulmonary arterial pressure; mmHg, millimeter of mercury; P/F: ratio of arterial oxygen partial pressure to fractional inspired oxygen; FEV1, forced expiratory volume in 1 s; FVC, forced vital capacity; DLCO, diffusing capacity of the lung for carbon monoxide; 6MWT, 6 min walking test; MRSA, methicillin-resistant *Staphylococcus aureus*; NTM, non-tuberculous *Mycobacteria*; PDE-5, phosphodiesterase type 5.

**Table 2:** Comparison of autonomic parameters evaluated by spectral analysis between healthy controls and patients in supine **(a)** and orthostatic position **(b)**.

	Healthy Controls *n* = 49	LTx List Patients *n* = 49	*p*
**(a) SUP**			
Heart rate, median (IQR) bpm	62 (58–71)	88 (79–99)	<0.0001
Spectral analysis, median (IQR)			
Total power, ms ^2^	2037 (1114–3706)	533 (180–1051)	<0.0001
LFnu	31 (22–43)	64 (36–82)	<0.0001
HFnu	66 (53–74)	21 (8–36)	<0.0001
LF/HF	0.46 (0.34–0.81)	2.81 (1.30–9.00)	<0.0001
RR-RESP HFk ^2^	0.86 (0.77–0.92)	0.85 (0.50–0.93)	0.26
RESP HF, Hz	0.25 (0.22–0.30)	0.34 (0.26–0.39)	<0.0001
**(b) ∆ORT**			
Heart rate, median ∆ORT (IQR) %	22 (9–38)	12 (9–16)	<0.0001
Spectral analysis, median ∆ORT (IQR) %			
Total power	−31 (−56–22)	−23 (−56–42)	0.51
LFnu	105 (49–274)	5 (−20–33)	<0.0001
HFnu	−57 (−77–−39)	−1 (−55–50)	<0.0001
LF/HF	438 (204–1546)	9 (−60–188)	<0.0001

*n*, number; LTx, transplant list; IQR 25–75, interquartile range; SUP, supine; bpm, beats per minute; ms^2^, milliseconds^2^; LF, low frequency; HF, high frequency; nu, normalized; LF/HF, sympatho–vagal balance; RR, R-R interval; RESP, respiratory; K^2^, coherence; Hz, Hertz; ∆, delta; ORT, orthostatism.

**Table 3:** Autonomic parameters evaluated by spectral analysis of patients on the waiting list for lung transplant stratified by Lung Allocation Score (LAS). Comparisons between patients with LAS≤ and > than the median **(a)** and between patients with LAS≤ and > than the 75th percentile **(b)**.

	(a)			(b)		
	LAS ≤ Median *n* = 25	LAS > Median *n* = 24	*p*	LAS ≤ 75th p. *n* = 37	LAS > 75th p. *n* = 12	*p*
Heart rate, mean (SD) bpm	87 (± 18)	87 (± 15)	0.97	86 (± 17)	88 (± 12)	0.79
Spectral analysis, median (IQR)						
Total power, ms ^2^	597 (232–1091)	331 (150–928)	0.43	551 (206–1080)	439 (164–841)	0.72
LFnu	75 (50–85)	55 (28–69)	0.04	68 (52–85)	41 (22–62)	0.005
HFnu	14 (7–34)	25 (8–37)	0.36	14 (7–36)	25 (13–46)	0.37
LF/HF	5.98 (1.53–10.89)	2.24 (1.09–4.29)	0.17	4.13 (1.56–10.92)	1.70 (0.63–3.47)	0.04
RR-RESP HFk ^2^	0.73 (0.35–0.89)	0.90 (0.63–0.94)	0.11	0.80 (0.41–0.92)	0.91 (0.64–0.94)	0.35
RESP HF, Hz	0.35 (0.25–0.44)	0.34 (0.28–0.37)	0.67	0.35 (0.25–0.40)	0.34 (0.29–0.36)	0.91

*n*, number; p., percentile; IQR 25–75, interquartile range; bpm, beats per minute; ms^2^, milliseconds^2^; LF, low frequency; HF, high frequency; nu, normalized; LF/HF, sympatho–vagal balance; RR, R-R interval; RESP, respiratory; K^2^, coherence; Hz, Hertz.

## Supplementary Materials

The following are available online at https://www.mdpi.com/2077-0383/9/4/1146/s1, Table S1: Comparison of autonomic parameters evaluated by symbolic analysis between patients affected by cystic fibrosis (CF) and patients affected by other diseases (non-CF). Table S2: Comparison of autonomic parameters evaluated by symbolic analysis between patients affected by CF and patients affected by other diseases (non-CF); only patients with a LAS lower than the median LAS of the whole sample are considered. Table S3: Comparison of autonomic parameters evaluated by symbolic analysis between patients affected by CF and patients affected by other diseases (non-CF); only patients with a LAS higher than the median LAS of the whole sample are considered.
