# Supplementary material for: Sympatho–Vagal Dysfunction in Patients with End-Stage Lung Disease Awaiting Lung Transplantation
Source: J Clin Med. 2020 Apr 17;9(4):1146. doi: 10.3390/jcm9041146 (PMC7230240; doi:10.3390/jcm9041146)
Supplement: Supplementary file 1 [file jcm-09-01146-s001.pdf]

Table S1. Comparison of autonomic parameters evaluated by symbolic analysis between patients affected by cystic fibrosis (CF) and patients affected by other diseases (non-CF).

	CF<br>n = 32	non-CF<br>n = 17	*p*
**0V%, mean (SD)**	23 (± 15)	30 (± 16)	0.129
**2LV%, median (IQR)**	10 (4 - 14)	6 (2 - 7)	0.06
**2UV%, median (IQR)**	18 (9 - 27)	22 (12 - 25)	0.5

CF, cystic fibrosis; non-CF, affected by other pathologies; n, number; SD, standard deviation; IQR 25-75, interquartile range.

Table S2. Comparison of autonomic parameters evaluated by symbolic analysis between patients affected by cystic fibrosis (CF) and affected by other diseases (non-CF); only patients with a LAS lower than the median LAS of the whole sample are considered.

	CF<br>n = 16	non-CF<br>n = 9	*p*
**0V%, mean (SD)**	28 (± 16)	36 (± 11)	0.182
**2LV%, median (SD)**	8 (± 5)	4 (± 2)	0.052
**2UV%, median (SD)**	17 (± 11)	18 (± 7)	0.926

CF, cystic fibrosis; non-CF, affected by other pathologies; n, number; SD, standard deviation.

Table S3. Comparison of autonomic parameters evaluated by symbolic analysis between patients affected by cystic fibrosis (CF) and affected by other diseases (non-CF); only patients with a LAS higher than the median LAS of the whole sample are considered.

	CF<br>n = 16	non-CF<br>n = 8	*p*
**0V%, mean (SD)**	23 (± 17)	14 (± 11)	0.174
**2LV%, median (SD)**	11 (± 7)	13 (± 9)	0.62
**2UV%, median (IQR)**	18 (9 - 25)	25 (18 - 29)	0.17

CF, cystic fibrosis; non-CF, affected by other pathologies; n, number; SD, standard deviation; IQR 25-75, interquartile range.
